# Bearing Fruit

**Published:** 1902-10

**Authors:** 


### BEARING FRUIT.

**The State Medical Association's Campaign of Education.**

It will be very gratifying to the physicians of Texas—to the State Medical Association especially—to know that the seeds recently sown by them have fallen, some in good soil—and have begun to bear fruit. The committee appointed for the purpose of disseminating certain papers read at the Dallas meeting bearing on the subject of needed sanitary legislation in the interest of the public health printed and mailed 7500 copies in pamphlet form. A lively interest has been awakened in the subject, and especially in the necessity of measures to prevent the spread of the deadly white plague. The means recommended are, primarily, corralling the straggling indigent consumptives, who every winter flock to Texas, by putting them in a State consumptive hospital,—"retiring them from circulation"; and the disinfection of all railroad trains and all hotels and boarding houses. I know of at least four members of the coming Legislature who intend to draw up bills for a State hospital for indigent consumptives, and earnestly champion it before the Legislature. I am gratified to say that the chances of securing this much desired and badly needed reform are excellent. The public are awakening to the need of it, are scared up, and should be protected. Especially at this season are consumptives flocking into Texas, attracted by its mild climate and well known salubrity. It is as much as a man's life is worth to go into a sleeper or into one of the rooms of a hotel where the carpets and curtains and bedding are perhaps infected with the germs of—well, say, *itch*—("Think of it, dissolate man"; see Tom Hood), or scarlet fever in the scaly stage, or smallpox—ugh—or consumption—even if the sheets have (may be) been changed since the patient got out of the bed. I want to say right here, however, that unless somebody or some community will donate a site for a State hospital for consumptives, it will be very difficult to get an appropriation through. The site being granted, we will have no difficulty in getting the bill through with appropriation sufficient to build and equip such hospital. I suggest San Angelo, or Comfort or Kerrville as being ideal localities for it.

